# Evaluation of a web-based randomized controlled trial educational intervention based on media literacy on preventing substance abuse among college students, applying the integrated social marketing approach: a study protocol

**DOI:** 10.1186/s13063-022-06913-6

**Published:** 2022-12-12

**Authors:** Hanieh Jormand, Saeed Bashirian, Majid Barati, Forouzan Rezapur-Shahkolai, Mohammad Babamiri

**Affiliations:** 1 Vice chancellor for research and technology, Hamadan University of Medical Sciences, Hamadan, Iran; 2 Department of Public Health, School of Health and Autism Spectrum Disorders Research Center, Hamadan University of Medical Sciences, Hamadan, IR Iran; 3 Department of Public Health, School of Public Health and Research Center for Health Sciences, Hamadan University of Medical Sciences, Hamadan, Iran; 4 Department of Ergonomics, School of Public Health and Research Center for Health Sciences, School of Public Health, Hamadan University of Medical Sciences, Hamadan, Iran

**Keywords:** Substance abuse, Social marketing, Randomized controlled trial, Media literacy education, youth, Web-based program

## Abstract

**Background:**

Substance abuse is the actual psychosocial harm, especially in young people confronted with content marketing in nowadays media environment, a risk factor for experiencing substance abuse. Based on the literature review, education designed based on the cognitive-behavioral model and planning models, such as using a social marketing framework, is the most effective method to prevent addictive substance abuse. Also, media literacy related to substance abuse and the prototype willingness model is considered a new integrated approach to present the intervention measures’ desired results.

**Methods:**

The present study evaluates an intervention program based on media literacy on substance abuse prevention among students using an integrated social marketing approach. This study aims to complete the SMART model’s sixth and seventh stages, implementing intervention and evaluation. Participants will be students of Hamadan University, Iran. Randomization will occur at the university and school levels, and gathered data will appear at two-time (i.e., pre-test and three months follow-up). Intervention group students will obtain both substance abuse prevention education and substance abuse media literacy (SAML) education between pre-test and 3 months post-test. Students in the delayed intervention will be given this education after study accomplishment; this group will receive their regular courses except for substance abuse prevention and media literacy titles during education intervention. The outcome variables are intentions and substance abuse behavior based on prototype willingness and substance abuse media literacy.

**Discussion:**

Evaluation of Substance abuse prevention and substance abuse media literacy education must be careful to ensure that they effectively enable people, especially in youth in the new media ecology and unique “Infomedia” ecosystems, in the current digital society. The SAML education plan’s evaluation has the first web-based education program in universities. No prior research has psychometrically considered SAML in students in the SMART model’s sixth and seventh stages.

**Trial registration:**

IRCT20200914048719N1. Registered on June 30, 2021.

## Background

According to the United Nations Office on Drugs and Crime (UNODC), in 2017, a total of 271 million people aged 15 to 64 years were drug abusers [1, 2]. In this regard, Iran mainly deals with this social, political, and global problem due to its young population and geographical location [3]. Epidemiological studies suggest an average age of 17.8 ± 3.9 years for the onset of the first use of addictive drugs, which gradually increases with age. This age group is associated with young people’s entry into universities and starting the studentship period. The first year in a university is considered the most challenging time for students. This period includes social challenges of being away from families, meeting new friends with different cultures, and mental challenges (such as successfully performing multiple assignments). In turn, it can lead to mental disorders (such as depression) and increase the possibility of addictive drug abuse, loneliness, and estrangement [4].

On the other hand, previous studies have reported that young people are the most popular social media platform users [5, 6]. Also, the media have become a global culture in youth’s lives. These new opportunities facilitate access to social interaction and learning [7] while exposing them to environmental risk factors for youth’s health, including having a sedentary lifestyle [8] or lower life satisfaction [9], anxiety, sleep disturbance, [10], and stress [11]. The amount of time spent on the media is associated with addictive substances. The likelihood of substance abuse increases with exposure to images and media content on substance abuse, such as movies and clips [12–14]. In this regard, understanding the harms and functions of the media and cyberspace and acquiring media literacy and learning skills for critical analysis of media messages are the most essential and useful requirements to prevent risky behaviors such as substance abuse, smoking, and unsafe sexual activity [15].

Education must be moral and based on defined theories and models to achieve useful and practical results. Among the ideas and models that are systematically used as a framework and roadmap for the educational process and goals to change behavior are planning models. The social marketing approach, in addition to analyzing successes and failures, provides the necessary guidance for reviewing and educational diagnosis. It also provides educational planning and intervention design methods, facilitates evaluation, and reduces the confusion of educational intervention designers [16]. Dann sees social marketing to promote positive behaviors and abandon negative behaviors [17, 18]. According to the 2002 definition, social marketing means “the customer-centric application of marketing principles and techniques to the development of programs, interventions, and evaluations to change or modify health behavior” [17–19].

Social marketing is a strategy to correct social behaviors. The 2 education strategies and coercion can be used to promote healthy actions and correct high-risk behaviors, including substance abuse.

The social marketing model was selected as a conceptual framework and appropriate research planning by reviewing the patterns and theories of changing individual and collective behavior. Social marketing has been used in various studies to influence various topics (such as tobacco prevention and promoting smoking cessation and alcohol reduction in women and students) [17, 20] and health problems (such as addiction and substance abuse) [20].

**Fig. 1:**
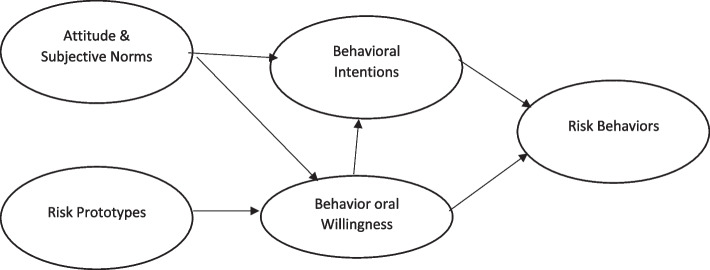
The PWM model

The prototype willingness model (PWM) is a model reflected in substance abuse, tobacco, and health risk decision-making, especially in youth and adolescents. PMW proposes behavioral intention, an intermediate variable named behavioral willingness. Behavioral willingness is measured by this essential question “how they react to risky behaviors in a certain social condition.” Besides intention and behavioral willingness, this model contains other variables. When individuals have a positive attitude toward high-risk decision-making and are more attracted to essential factors, they will be more willing to carry out dangerous behavior. According to PWM, individuals have prototypes that play a role in high-risk behaviors (Fig. 1) [21].

Media literacy will be added to PWM as a background factor. It will be examined as extended PWM; thus, the media is a background factor in forming the positive and negative prototypes of youth and adolescents. Strong evidence suggests the role of social networking sites in alcohol use [22], showing that youth exposed to alcohol media content on social networking sites increase their alcohol consumption, engage in heavy drinking, and show positive attitudes toward alcohol use [23]. In a similar vein, studies have found that 25–37% of youth post details about their alcohol drinking, which may give younger adults and adolescents the impression that substance abuse is a common behavior among peers of the same age [24, 25]. Accordingly, regarding the prevalence of substance abuse in universities and its negative consequences [26] (e.g., decreased academic motivation, academic failure, physical and mental illness, suicide), as well as conducting a comprehensive study in this field and identifying factors influencing the tendency to use drugs in students, this study’s main objective was to evaluate the effectiveness of substance abuse media literacy (SAML) education and substance abuse education prevention in students. This media literacy intervention was intended to provide and develop skills to analyze and interpret risky content in social media apps (such as WhatsApp, Telegram, and Instagram) and social networking sites because this new medium may also overrepresent media messages about substance abuse [27–30].

### Social media literacy concerning substance misuse and substance abuse media literacy

Social media literacy and SAML concepts have recently emerged as a distinct sub-discipline of media literacy. As a result of the interactive nature of SNSs, these concepts of media literacy include the capacity to reflect on one’s online behavior [31, 32].

Notably, SAML has four dimensions (Purpose, Constructed-ness, High sensation-seeking audiences, Format, Filter & omit) [27, 29] which are different from the content dimensions of social media literacy: the self, medium, and reality [33].

Both of these concepts demonstrate the ability to locate individuals in the connections between the media and actual worlds, be conscious of the exchanges between the self and the media, and distinguish the values of these exchanges [33].

On the other hand, substance abuse media literacy is the skill to analyze and interpret substance use content in social media apps; this skill prevents youth from encouraging them to develop unhealthy tendencies concerning substance use [32]. Both concepts include SAML and social media literacy have the same goal; empowerment of youth to new media risky contents encounter but with different content dimensions.

The study will also assess process evaluation. However, no information is available about aspects of media literacy about substance abuse in Iran. Therefore, this study aimed to evaluate an intervention program based on media literacy on preventing substance abuse among students using an integrated approach to social marketing. Therefore, a randomized, controlled clinical trial study with a parallel-group design of students was performed in the present study.

## Methods/design

### Aim, design, and setting

The study design evaluated web-based randomized controlled trial substance abuse prevention and SAML education programs. It was expected that 3 universities (including Hamadan University of Medical Sciences, Hamadan Islamic Azad University, and Bu-Ali Sina University) would take part in the study. Data were collected from students at 2-time points (i.e., pretest and 3-month follow-up). Interventional research was based on the Social Marketing Assessment and Response Tool (SMART) pattern.

**Fig. 2:**
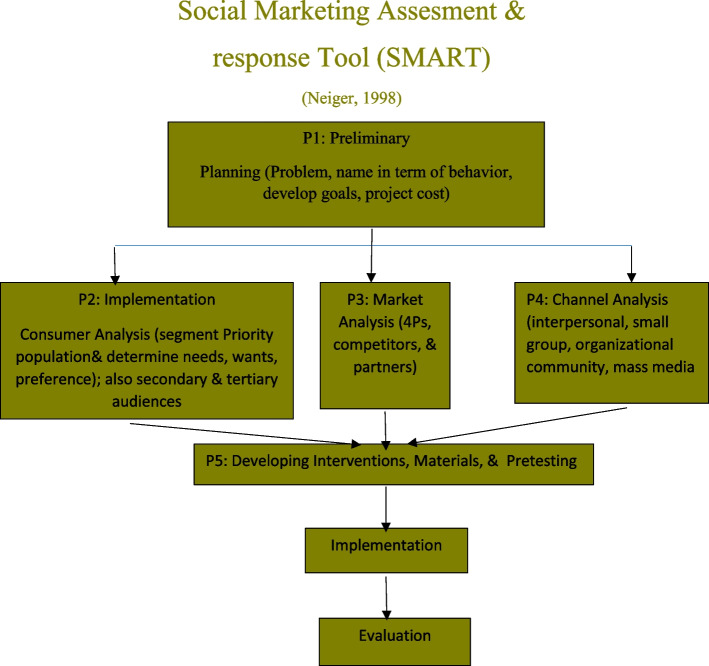
SMART

SMART is the operational model of social marketing in the field of health, which is used to improve the behavior of society and includes 4 stages as follows: (1) preliminary planning, (2) formative research (customer analysis, market analysis, and channel analysis), (3) production of interventions, materials, and pretests, and (4) intervention and execution (Fig. 2).

The above outcomes will be obtained in the experimental study.

It should be noted that in this model, previous formative research (including qualitative and descriptive studies) was performed to complete steps 1 to 5 of the SMART model to determine the type of intervention and products (including getting detailed information about audience peculiarities, behavioral market, and communication channel by audience analysis, market analysis, and channel analysis). In a qualitative study, the audience needs and desires were identified, and behavioral market determinants (such as partners and components of the marketing mix and channels) were specified. An effective communication strategy was developed, which was the result of this study. Notably, based on this previous formative-mixed methods research, the substance abuse prevention intervention was considered more accurate, and this intervention was developed based on the most prevalent addictive substances in college students. Also, media literacy intervention would benefit from greater detail based on this previous formative research.

Here are 3 questions to which formative research provided answers: (1) what is the most commonly used substance on college campuses? (2) Which university has a higher prevalence of drug abuse? (3) Which behavioral and non-behavioral factors are involved in the health problem? To answer these questions in the descriptive study, which is a quantitative part of the formative research, students’ demographic information and drug use behavior were assessed based on PWM and media literacy related to substance abuse. The analysis of the qualitative and quantitative parts of the formative research identified the main components of the intervention.

Therefore, based on the results of this formative research, the most commonly used substance on college campuses was marijuana in Hamadan universities. Also, behavioral and non-behavioral factors involved in substance abuse behavior (such as the role of parents, friends, and SAML on risky behaviors) were identified.

Notably, this study (protocol study) was performed to complete the SMART model’s sixth and seventh stages, including the implementation of intervention and evaluation.

It was anticipated that up to 116 students might take part in the study. This study would have taken place during the 2020–2021 school year. Additionally, program completion and feedback on process evaluation (satisfaction, availability, and quality of material education) would be gathered from the intervention students. The research team used the Standard Protocol Items: Recommendations for Interventional Trials (SPIRIT) checklist to prepare this study [34].

### Sample size and eligibility criteria

According to previous studies, the intervention’s minimum effectiveness is 20% [35–37], referring to changes in the PMW construct, behavior, and SAML. Also, because of considering these cultural differences in the intervention’s minimum level of effectiveness, 20% was considered for intervention effectiveness based on the previous study with the same field and approach; taking into account the clustering coefficient of 1.5, alpha 5%, and 90% desired power, we need 54 people per group. Also, considering the 20% probability of attrition in the intervention process, the present study’s sample size was 116, including 58 in the experimental group and 58 in the control group.

$$n=\frac{\overline{p}\left(1-\overline{p}\right){\left({Z}_{\beta }+{Z}_{\alpha /2}\right)}^2}{{\left({p}_1-{p}_2\right)}^2}$$

Inclusion criteria were access to the internet and social media apps (such as WhatsApp, Telegram, and Instagram), being a student in one of the universities in the city of Hamadan, and willing to participate in the study. Before participation, students signed written informed consent forms to participate in this study. The Principal Investigator will access consent forms of student participant data that will be nameless. Also, according to the consent form, students were free to withdraw from the study at any time.

### Internal review board approval

This paper was taken from a doctoral dissertation in Health Education and Health Promotion. This study was approved by the Research Ethics Committee of Hamadan University of Medical Sciences (code: 9811018459; ID: IR.UMSHA.REC.1398.827).

### Participant characteristics

Subjects were enrolled in this study in 2 plans: university schools and students. It should be noted that Hamadan city is located in the west of Iran. It has 4 state universities, including Bu-Ali Sina University, Hamadan University of Medical Sciences, Hamadan University of Applied Sciences and Technology, and the Hamadan University of Technology. Moreover, it has some non-governmental universities, such as Hamadan Islamic Azad University and Hamadan Payame Noor University [38]. Students were selected by simple random sampling using their ID codes. Thus, students in the cluster in the university, once they are categorized into their stratum, then they are randomized to either the intervention or the control groups.

### Processes

#### Schools of university

First, university permission to conduct the study was obtained. University administrators would access questionnaires for an estimate if demanded. Efforts were made to balance the inclusion of both state and non-state universities, as well as to range in their socio-demographic characteristics. Based on the previous formative research [27] and random sampling of Hamadan universities, 3 universities were expected to participate. Therefore, the number of students in each school or university was obtained by proportional assignment of students to the school (i.e., the school with the largest number of students, the largest number of students assigned). After coordinating with university officials, the research units were rationed. Then, the samples were randomly collected.

#### Students

It was anticipated that up to 161 students might be enrolled in this study. Research team members explained the aims of the survey to students and distributed consent forms. Students were eligible to participate if they were members of Hamadan universities and consented to receive SAML and substance abuse prevention education. Students completed consent and permission forms, respectively, before initiating the study.

#### Protocol

After the Research Ethics Committee of Hamadan University of Medical Sciences approval, the Principal Investigator shared a copy of the revised protocol with all the research team members to ensure awareness of the reconsiderations. The research team will document any unexpected deviations from the protocol. Concerning the Data and Safety Monitoring Plan, the Research Ethics Committee of Hamadan University of Medical Sciences will be informed of any updates and modifications.

#### Randomization

Randomization was performed using a stratified random sampling procedure; individuals were assigned to 2 intervention and control groups. Randomization was performed after students signed informed consent, as well as the approval of universities to perform the study.

#### The intervention description

As noted based on the previous formative-mixed methods research, materials, the content of interventions, and the type of delivery channel to participants (which was based on audience tendencies) were identified and developed, and interventional education was developed by an interdisciplinary group consisting of specialists in psychology, media research, web site development, multimedia design, and health education and promotion.

SAML and preventive substance abuse educations were web-based and comprehensive education programs for university students, providing appropriate and medically accurate information about substance abuse using a media literacy education approach. It should be noted that this interventional education would be web-based because of the COVID-19 pandemic to comply with health protocols in Iran. Some advantages of this approach are as follows: Because of the COVID-19 pandemic and social-distancing measures in Iran, it is not clear when universities can return to normal education situations and face-to-face educational interventions. Thus, a web-based approach to delivering education interventions in person was selected. Also, this approach allows for a fully interactive environment or 2-way forms of communication, often between individuals who know each other (e.g., friends) and may have a strong influence on behavior. Moreover, the health of participants is valuable and important for all researchers.

Also, the web-based channel was based on audience preferences and provided more access to materials for interventions. This channel is more flexible, and students can access it at all times and any place, and any individual concerning personal conditions can educate and use educational materials.

Some disadvantages of this approach are as follows: access to comprehensive materials and support can be difficult, and this approach requires additional training before starting educational interventions.

The pairing of preventive substance abuse information and SAML education allows the program to teach students critically thinking about media messages related to substance abuse specifically marijuana and substance abuse behaviors. It mostly teaches them what lifestyles, values, and points of view are represented in or omitted from media messages about substance abuse, such as identification and the possible implications of actual consumption of drugs, including simulating an actor, having instant excitement, believing in the non-addictive nature of advertised substance, and having ideal moods and herbal and not harmful effects.

**Fig. 3:**
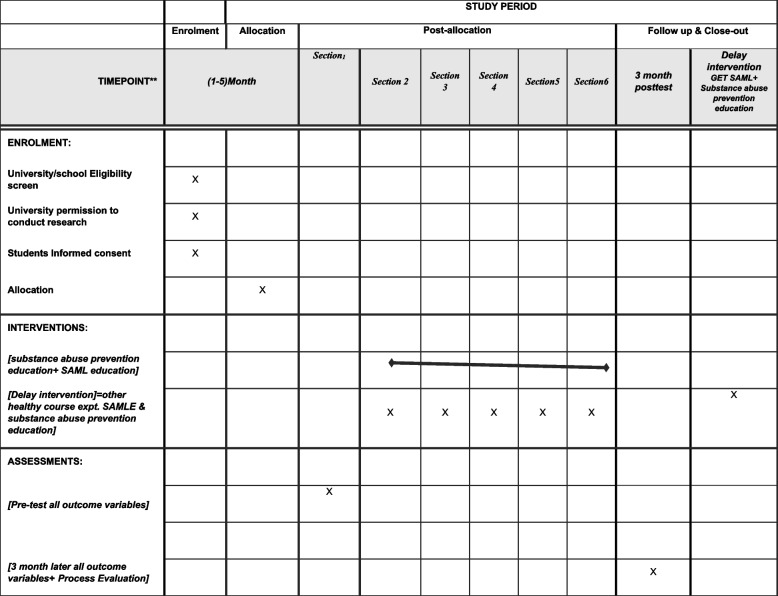
Template of SPIRIT recommended content for the schedule of enrolment, interventions, and assessments

Education consisted of 5 web-based education sessions about substance abuse prevention and SAML. Each session included 40–45 min of training with the question-and-answer methods. Group discussions (chat rooms) were held with multimedia tools and reminder messages, such as the first educational session to familiarize students with drugs, especially marijuana and substance abuse behavior consequences. The second session aimed to educate students on SAML skills, critical thinking, and media consumption regime. The third session aimed at familiarizing students with interpersonal skills and self-awareness, and the fourth session taught students how to say no and how to solve problems. The fifth session corrected students’ beliefs and attitudes toward substance abuse using the mentioned web-based teaching methods and materials—each containing group discussion in chat rooms. The intervention group received lessons between completing the pretest and follow-up (Fig. 3). The website contained an educator dashboard for educators. They will be one of the research teams to enroll the students in the program and group discussions, including chat rooms, to correct their beliefs about drugs. Educators were also allowed to use web-based education programs in order to make education programs more accessible to students. All educators were requested to review all of the materials.

Disincentive students received a gift card of $30 for participating in the study. Students who participated in all data gathering phases (2 times) received a gift card of $50 at follow-up. Students accessed SAML and substance abuse prevention education until the end of the semester.

#### Delayed-intervention group

Students in the delayed-intervention group received lessons during the study, but educators were requested to abstain from any substance abuse or SAML topics. Simultaneously, educators in the delayed-intervention group did not train them until after study completion (i.e., collecting student data at a 3-month follow-up).

#### Implementation procedure

As mentioned previously, participants in this study were familiar with e-learning classes and online education due to the COVID-19 pandemic. Educators accessed the web-based educator’s control panel, giving them access to programs. The principal investigator and the research team visited the website. Privacy shields were provided to block their computers or tablet screens from view to ensure confidentiality. Secret ID numbers were used in place of names in chat rooms, and during receiving education programs, admin and educator sessions were offered to students before education. Students were instructed that they could withdraw from the study at any time for any reason. Follow-up questionnaires were completed with paper copies of the pretest questionnaire. After completing the data collection, educators in the intervention group provided substance abuse prevention and SAML education over 3 weeks. It was predicted that the program could be completed in 5 45-min sessions. In the follow-up period, students in the intervention group gave their feedback on the program. Educators returned all completed follow-up questionnaires to administer and Principal Investigator after approximately 3 months. After collecting the follow-up data, educators in the delayed-intervention group could provide SAML and substance abuse prevention education to students and give feedback on their experiences.

### Measures

#### Student measures

Students completed questionnaires at 2-time points: a pretest and a 3-month follow-up (Fig. 1). The student questionnaire, containing approximately 100 items, covered substance abuse behavior based on PWM variables outcomes (e.g., attitudes, normative beliefs, behavioral intentions, and willingness), self-reports of the SAML Scale (SAMLS), and process evaluation. In addition, demographic questions comprised age, education level, socioeconomic status, sex, gender, parental education level, marital status, name of university, schools and courses, history of smoking and alcohol consumption, addiction status in parents, close friends, substance abuse status, internet access, amount of social media exposure and use of social media, and motivation to use social media.

#### Outcome measures

- Scales of substance abuse behavior based on PWM included the constructs of attitude, subjective norms, and behavioral willingness, as well as prototype and intents of behavior [32, 39]
- SAMLS includes 13 questions [29]
- Process evaluation checklist

Process evaluation included student satisfaction and availability of educational material. The quality of educational material was assessed using a 5-point Likert scale.

#### Plans to promote participant retention and complete follow-up

It should be noted that after obtaining informed consent, participants were randomly given ID numbers to be used in the education program. The list of names and ID numbers was confidentially kept on a secure server, only accessible to the principal investigator and administration staff (skilled in the ethical study). If any participant discontinued or withdrew from the education plan, administration staff would make efforts to encourage him/her to continue the study via 2 contacts.

#### Blinding description

Participants were divided into intervention and control groups (delayed-intervention group) after obtaining informed consent. Trials were planned so that participants were aware of all the aims of the study. Based on the research group’s planning, all members of the intervention and delayed-intervention groups accessed all education. The intervention group members were initially educated, and the members of the delayed-intervention group received training 3 months after receiving the post-test results. It is necessary to mention that students were unaware of assignments in the study groups.

#### Fidelity of implementation process

These educations started and ended based on research team planning. The real lessons of the online session were accessible to all students without any limitations.

#### Statistical methods

Data were analyzed with SPSS version 23 (SPSS Inc., Chicago, Ill., USA) using analysis of variance (ANOVA), independent *t*-test, and other tests. Generally, if the mean score of variables before the intervention was significantly different between the intervention and control groups, the ANOVA test was used; otherwise, we used an independent *t*-test. The independent samples *t*-test (or the Mann-Whitney *U* test) was used to determine differences in numerical variables between the intervention and control groups. To draw a within-group comparison, we used the paired-sample *t*-test or its nonparametric equivalent, the Wilcoxon test. If necessary, missing data were examined at each time point and handled with an appropriate imputation method. All data of the full protocol and intervention study were potentially shareable after unidentified individuals. All researchers could access the data upon request to the Vice-Chancellor for Research and Technology of Hamadan University of Medical Sciences.

#### Oversight and monitoring

As mentioned previously, this study was performed to complete the SMART model’s sixth and seventh stages, including the implementation of intervention and evaluation. Confidentiality was assured to all participants. Participants were randomly given ID numbers to be used in the education program. The list of names and ID numbers was confidentially kept on a secure server, only accessible to the principal investigator and administration staff (skilled in the ethical study). After completing the follow-up period, the data collection was completed. Students completed the number of answer questionnaires. Identifying information could not be shared by the students; thus, ID numbers were used in place of names in chat rooms and to receive education programs. As this study was a minimal-risk study, the occurrence of any adverse events was reported to the Principal Investigator and the Research Ethics Committee of Hamadan University of Medical Sciences to make the final decision on whether or not to continue the trial.

The possibility of serious adverse events was evaluated within 24 hours, and any other challenges were considered within 72 h, reporting to the Research Ethics Committee of Hamadan University of Medical Sciences within 2 weeks. The procedure of auditing was checked by the Principal Investigator 3 times, including drafting the proposal, conducting the study, and ending the study.

#### Ethics and dissemination

All university students were recruited for this study by the Research Ethics Committee of Hamadan University of Medical Sciences. All participants were voluntarily enrolled in this study. The researcher’s contact information was printed on the forms in case students had questions about the study. Also, this study was approved by the Research Ethics Committee of Hamadan University of Medical Sciences (code: 9811018459; ID: IR.UMSHA.REC.1398.827).

#### Sharing plan

All data is potentially shareable after unidentified individuals. All researchers could access the data upon request to the Vice-Chancellor for Research and Technology of Hamadan University of Medical Sciences. It is worth noting that results from this study will be prepared for publication in scholarly journals and updated on https://www.irct.ir/.

#### Confidentiality

As it was mentioned before, confidentiality was assured to all participants in all processes of the implementation procedure and monitored until the end of education programs. Participants were randomly given ID numbers to be used in the education programs. The list of names and ID numbers was confidentially kept on a secure server, only accessible to the Principal Investigator and administration staff (skilled in the ethical study). Also, the Principal Investigator checked the coding, security, and storage of data.

## Discussion

SAML and substance abuse prevention education programs must be carefully evaluated to ensure that they effectively enable people in youth health and media literacy skills, mainly about substance abuse behavior in the new media ecology and unique “Infomedia” ecosystem digital societies. To our knowledge, the SAML education programs being evaluated in this study can be the first web-based education program in universities; no prior research has psychometrically considered SAML in students who are among the high-risk groups regarding drug use. The study design, obviously including students’ data collection, allows for evaluation of the program’s immediate effects on student outcomes and any behavioral effects during the 3 months after the students receive the program. Also, gathering student feedback will help determine the chance of program implementation by universities.

We conclude that despite numerous advantages, internet-based educational intervention has some challenges; for instance, in the present study, the amount of time/response burden may be imposed on students. Concerning different effective factors and examining important constructs of PWM, the researchers had no options except for using a 100-item pre-post measure. Thus, this number of items could be considered a limitation. Therefore, web-based approaches may become an important new approach to reducing respondent burden. Also, it should be regarded that this intervention study will focus on specific social media and not traditional media channels. So, future study concerning all media platforms was recommended.

### Trial status

The protocol is Version 1, dated 6 October 2020. The trial is registered on the Iranian Registry of Clinical Trials, IRCT20200914048719N1, with the available title “Effect of an Educational Intervention Based on Media Literacy on Substance Abuse Prevention among College Students” and the scientific title “The Effect of Educational Intervention Based on Media Literacy on Substance Abuse Prevention among College Students Applying the Integrated Social Marketing Approach: a clinical trial study.” The recruitment date began on 5 November 2020; recruitment is scheduled to be finished by the end of May 2021. The problem of the study is substance abuse prevention in youth. The trial sponsor is Hamedan University of Medical Sciences with a number 9811018459.

## Data Availability

All data may be obtainable from the corresponding author.

## Abbreviations

SAML: Substance abuse media literacy
PMW: Prototype Willingness Model
